# SARS-CoV-2 spike downregulates tetherin to enhance viral spread

**DOI:** 10.1101/2021.01.06.425396

**Published:** 2021-01-06

**Authors:** H Stewart, KH Johansen, N McGovern, R Palmulli, GW Carnell, JL Heeney, K Okkenhaug, AE Firth, AA Peden, JR Edgar

**Affiliations:** 1 Department of Pathology, University of Cambridge, Cambridge. CB2 1QP. United Kingdom.; 2 Laboratory of Immune Systems Biology, National Institute of Allergy and Infectious Diseases, National Institutes of Health, Bethesda, United States of America.; 3 Department of Veterinary Medicine, University of Cambridge, Cambridge. CB3 0ES. United Kingdom.; 4 Department of Biomedical Science, Centre for Membrane Interactions and Dynamics, University of Sheffield, Firth Court, Sheffield. S10 2TN. United Kingdom.

## Abstract

The antiviral restriction factor, tetherin, blocks the release of several different families of enveloped viruses, including the *Coronaviridae*. Tetherin is an interferon-induced protein that forms parallel homodimers between the host cell and viral particles, linking viruses to the surface of infected cells and inhibiting their release. We demonstrate that SARS-CoV-2 downregulates tetherin to aid its release from cells, and investigate potential proteins involved in this process. Loss of tetherin from cells caused an increase in SARS-CoV-2 viral titre. We find SARS-CoV-2 spike protein to be responsible for tetherin downregulation, rather than ORF7a as previously described for the 2002–2003 SARS-CoV. We instead find ORF7a to be responsible for Golgi fragmentation, and expression of ORF7a in cells recapitulates Golgi fragmentation observed in SARS-CoV-2 infected cells.

## Introduction

The newly described coronavirus, severe acute respiratory syndrome coronavirus 2 (SARS-CoV-2) is the causative agent of coronavirus disease 2019 (COVID-19) (1, 2). Efforts to develop drugs and therapies remain challenging without a detailed molecular understanding of how SARS-CoV-2-infected cells give rise to COVID-19. Coronaviruses are positive-sense single stranded RNA viruses, and their genomes encode a large non-structural replicase complex and four main structural proteins, spike (S), membrane (M), envelope (E), and nucleocapsid (N). In addition, the viral genome encodes other accessory proteins that facilitate replication, cell entry and immune evasion.

SARS-CoV-2 cellular entry is mediated by spike protein binding to the host receptor angiotensin-converting enzyme-2 (ACE2) (3). Unlike that of SARS-CoV-1, the SARS-CoV-2 spike protein contains a polybasic furin cleavage site which facilitates the cleavage of the spike into two proteins, S1 and S2 that remain non-covalently associated (4, 5). The S2 fragment is further primed by the serine protease TMPRSS2 (3), whilst the S1 fragment binds Neuropilin-1 (6, 7), facilitating virus entry and infection. Following binding at the cell surface, coronaviruses are endocytosed to endosomes where their envelope fuses within late endosomes/lysosomes (8), liberating the viral capsid to the cytosol of the cell. Subsequently, viral proteins are translated and assembled at modified tubulovesicular ERGIC (endoplasmic reticulum-Golgi intermediate compartment) organelles. Coronaviruses modify host organelles to generate viral replication factories, so-called DMVs (double-membrane vesicles) that act as hubs for viral RNA synthesis (9).

Viruses can be broadly categorized by the presence or absence of a host-derived lipid envelope. Membrane envelopment protects the viral capsid from the external environment, reduces host immune recognition, and aids viral entry to new cells. However, this envelopment process does provide an opportunity for host cells to integrate anti-viral factors to the forming virions. A number of enveloped viruses become tethered at the plasma membrane of cells following their delivery to the cell surface, and this retention inhibits the viral spread and infection of naïve cells and is mediated by the host protein tetherin (*Bst2*).

Tetherin is a type II integral membrane protein with a short cytosolic tail, large extracellular coiled-coil domain that is anchored to the membrane via a C terminal GPI anchor. It is constitutively expressed by many cell types but is upregulated in the presence of type-I interferon (10). Cysteine residues in the extracellular loop mediate homodimer formation, linking tetherin molecules on virus and cell membrane, leading to the retention of nascent viral particles on the surface of infected cells (11). The cell surface retention of virions enhances their reinternalization to endosomal compartments, limiting the extent of virus spread (12).

Enveloped viruses including human immunodeficiency virus 1 (*Lentivirus*) (10, 13), Ebola virus (*Ebolavirus*)(10, 14), Kaposi’s sarcoma-associated herpesvirus (KSHV) (*Rhadinovirus*) (15) and human coronavirus 229E (HCoV-229E) (*Alphacoronavirus*) (16) undergo tetherin-dependent restriction. For enveloped viruses to produce fully released progeny, they have evolved means to counteract tetherin activity. Although the molecular mechanisms by which each virus downregulates tetherin differs, their outcomes converge resulting in loss of tetherin from the plasma membrane and enhanced virion release.

Of the previously described coronaviruses, HCoV-229E and 2002–2003 SARS-CoV (hereafter named SARS-CoV-1) have been shown to undergo viral restriction by tetherin(16, 17). Two SARS-CoV-1 proteins have been shown to downregulate tetherin resulting in a concomitant increase in virion spread – the ORF7a protein and S (spike glycoprotein)(17, 18). However, several questions remain about the mechanisms surrounding tetherin downregulation in coronaviruses. It is also unclear exactly how and where tetherin forms such tethers, as in coronaviruses, unlike other viruses that undergo tetherin-dependent restriction, membrane envelopment occurs in the biosynthetic pathway and not at the plasma membrane. Both *ORF7a* and *Spike* genes differ between SARS-CoV-1 and SARS-CoV-2, with ORF7a protein containing 85% and spike 76% sequence identity (at the amino acid level). Whether either of these proteins downregulate tetherin for SARS-CoV-2 remains unknown.

Here, we show that tetherin is directly responsible for tethering of nascent enveloped SARS-CoV-2 virions to infected cell surfaces. Infection of cells with SARS-CoV-2 virus causes a dramatic downregulation of tetherin from the cell surface. We investigate two proteins that were previously described to downregulate tetherin during SARS-CoV-1 infection – the ORF7a protein and spike. We show that SARs-CoV-2 spike is responsible for tetherin downregulation whereas ORF7a protein causes fragmentation of the Golgi.

## Results

**Figure 1 –:**
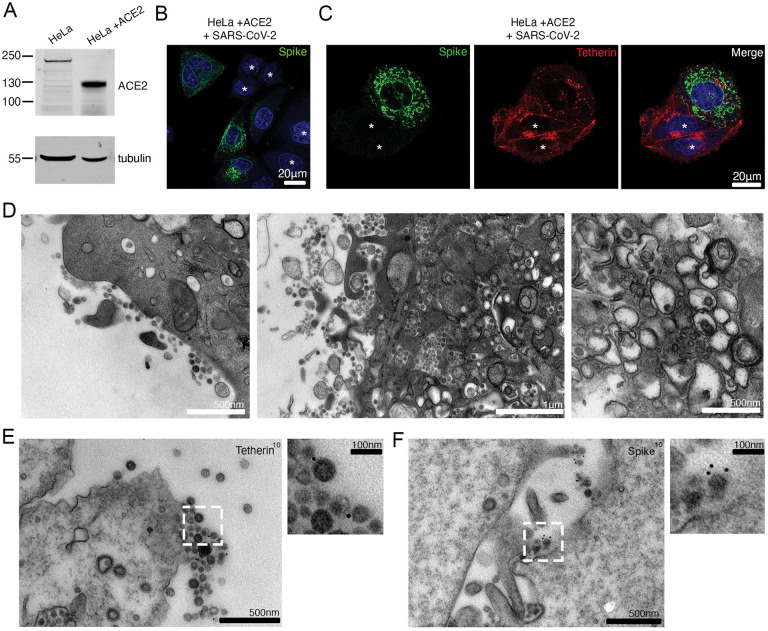
SARS-CoV-2 infection downregulates tetherin in HeLa^WT^ +ACE2 cells. (A) HeLa cells were transduced with ACE2 lentivirus to generate stable cell lines. Mock and ACE2 transduced cells were lysed and immunoblotted for ACE2. Tubulin was used as a loading control. (B) HeLa^WT^ +ACE2 cells were infected with SARS-CoV-2 (MOI 0.5). Cells were fixed at 24 hpi and stained for spike (green) and DAPI (blue). (C) HeLa^WT^ +ACE2 cells were infected with SARS-CoV-2 (MOI 0.5). Cells were fixed at 24 hpi and stained for spike (green), tetherin (red) and DAPI (blue). Uninfected cells shown with asterisk. (D) Electron micrographs showing plasma membrane associated SARS-CoV-2 virions and virus filled intracellular organelles. SARS-CoV-2 infected HeLa^WT^ +ACE2 cells (MOI 0.5) were fixed at 24 hpi and processed for TEM. Left micrograph – plasma membrane-associated virus, middle micrograph – virus-filled tubulovesicular compartments are directed towards the plasma membrane, right micrograph – virions within DMVs. (E) Surface immunogold electron microscopy of SARS-CoV-2 infected HeLa^WT^ +ACE2 cells. Cells were infected with SARS-CoV-2 (MOI 0.5), fixed at 24 hpi and immunogold labelled with antibodies against tetherin. (F) As (E) but labelled with antibodies against SARS-CoV-2 spike.

To establish whether SARS-Cov-2 downregulates tetherin, we first generated a HeLa cell line stably expressing ACE2. HeLa cells express abundant levels of tetherin at steady state, but do not express ACE2 endogenously. ACE2 stable HeLa cells, designated as HeLa^WT^+ACE2 were generated by lentiviral transduction and ACE2 protein expression was confirmed by Western blotting (Figure 1A). Using a clinical isolate of SARS-CoV-2 (isolate BetaCoV/ Australia/VIC01/2020) (19), we performed viral infection assays and fixed the cells 24 hours post infection (hpi). Infected cells were confirmed by spike labelling (Figure 1B, uninfected cells shown with asterisk).

Uninfected HeLa^WT^+ACE2 cells display tetherin localised to the plasma membrane and to intracellular perinuclear compartments, whereas infected cells display a loss of tetherin from the plasma membrane and an increase in intracellular punctate staining (Figure 1C, Supplemental Figure 1A) (uninfected cells shown with asterisk). While tetherin appeared to be broadly downregulated from the plasma membrane, some plasma membrane staining remained in these cells that often colocalised with spike labelling (Supplemental Figure 1A), likely representing areas of surface-tethered SARS-CoV-2 virus.

To examine whether SARS-CoV-2 virions were tethered to the cell surface, we performed transmission electron microscopy. In infected HeLa^WT^+ACE2 cells, SARS-CoV-2 virions could be found clustered on the plasma membrane of cells, although tethered virions were frequently polarised to discrete areas, rather than distributed evenly along the plasma membrane (Figure 1D, Supplemental Figure 1A). Virus-containing tubulovesicular organelles were often polarised towards sites of significant surface-associated virus.

Electron microscopy also verified the presence of double membrane vesicles (DMVs) in infected cells, and typical Golgi cisternae were not present in infected cells (Figure 1D). To check whether the formation of DMVs and aberration to the biosynthetic machinery was causing a global downregulation of surface proteins, we stained infected cells for the surface protein beta2microglobulin (Supplemental Figure 1B), but no obvious loss in plasma membrane staining was observed in infected cells. This is consistent with other reports demonstrating specific proteins are up- or downregulated (20). Surface labelling immunogold electron microscopy (see Methods) revealed tetherin molecules to be found between SARS-CoV-2 virions (Figure 1E) and virions were verified as being SARS-CoV-2 using an anti-SARS-CoV-2 spike antibody (Figure 1F).

**Figure 2 –:**
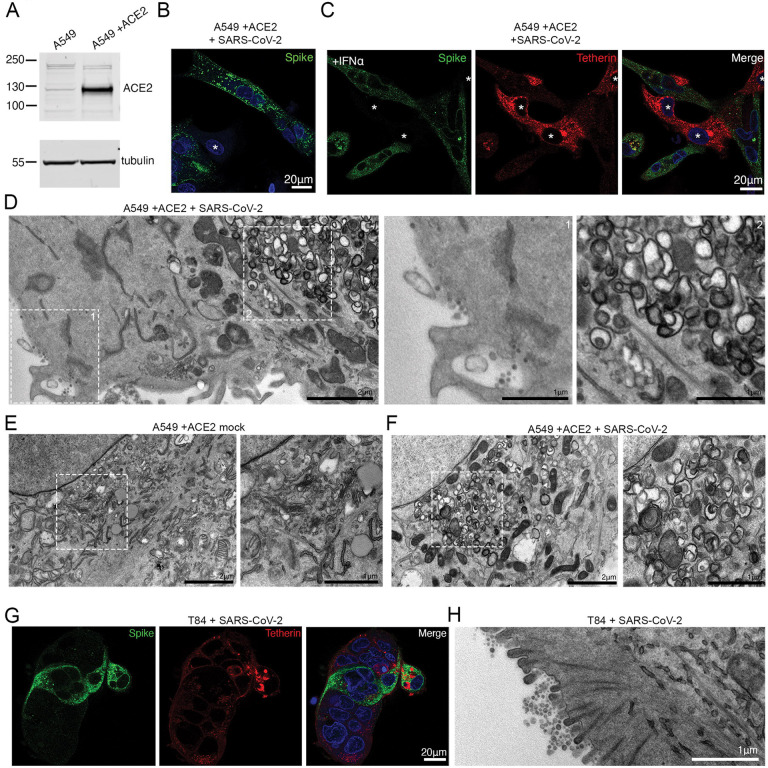
SARS-CoV-2 infection of A549 and T84 cells. (A) A549 cells were transduced with ACE2 lentivirus to generate stable cell lines. Mock and ACE2 transduced cells were lysed and immunoblotted for ACE2. Tubulin served as a loading control. (B) A549+ACE2 cells were infected with SARS-CoV-2 (MOI 0.5). Cells were fixed at 24 hpi and stained for spike (green) and DAPI (blue). Uninfected cells shown with asterisk (C) A549+ACE2 cells were treated with IFNα (1000 U/ml, 24 hours) to upregulate tetherin expression. Cells were infected with SARS-CoV-2 (MOI 0.5). Cells were fixed at 24 hpi and stained for spike (green), tetherin (red) and DAPI (blue). Uninfected cells shown with asterisk. (D) A549+ACE2 cells were treated with IFNα (1000 U/ml) and infected with SARS-CoV-2 (MOI 0.5), fixed at 24 hpi and processed for TEM. Infected cells display very few virions on their plasma membrane (left inset) but significant DMV formation (right inset). (E) Electron micrograph of the perinuclear region of A549+ACE2 mock infected cells. Zoomed area shows typical Golgi morphology. (F) Electron microscopy of the perinuclear region of SARS-CoV-2 infected A549+ACE2 cells. Cells were infected at an MOI of 0.5 and fixed at 24 hpi. Zoomed area shows membrane rearrangements and DMVs. (G) T84 cells were infected with SARS-CoV-2 (MOI 0.5) and fixed at 24 hpi and stained for spike (green) and tetherin (red). (H) T84 cells were infected with SARS-CoV-2 (MOI 0.5) and fixed at 24 hpi and processed for TEM. Tethered virions were frequently present at the plasma membrane.

SARS-CoV-2 primarily infects respiratory epithelial cells. The human alveolar basal epithelial cell line, A549, express low levels of ACE2 endogenously, although overexpression of ACE2 can facilitate betacoronavirus entry (21, 22). A549 cells expressing ACE2, designated A549+ACE2, were generated by lentiviral transduction (Figure 2A) and these cells were amenable to SARS-CoV-2 infection (Figure 2B, uninfected cells shown with asterisk). A549 cells do not express tetherin at steady state, although its expression can be induced through stimulation with interferon alpha (IFNα)(10, 23) (Supplemental Figure 2A), revealing tetherin to be localised to the plasma membrane and to intracellular compartments. Immunofluorescence analysis of A549+ACE2 cells infected with SARS-CoV-2 revealed a dramatic loss of tetherin as revealed by the loss of tetherin (red) in spike positive (green) cells (Figure 2C, uninfected cells shown with asterisk). To examine what effect this near-total loss of tetherin had on virus tethering, we again performed electron microscopy. Infected, IFNα treated A549+ACE2 cells presented significant intracellular remodelling, but very few surface-associated virions were present, likely due to the significant tetherin downregulation (Figure 2D). Virion-containing DMVs were frequently observed in the perinuclear region of infected cells, and these were associated with dramatic membrane remodelling, including a loss of typical Golgi cisternae from cells (Figures 2E, 2F).

The human colonic adenocarcinoma epithelial cell line, T84, which expresses endogenous ACE2 (24) were examined for their ability to be infected by SARS-CoV-2 and to tether virions (Figure 2G). Electron microscopy analysis of infected T84 cells displayed significant virus tethering at microvilli (Figure 2H), along flat regions of the plasma membrane, and formation of virus-filled intracellular compartments (Supplemental Figure 2B). The differences in the amount of tetherin downregulation between these cell lines may reflect either differences in kinetics of infection, differences in resting levels of tetherin, or differences in host machinery involved in the process of downregulation. These data are consistent with previous observations suggesting that other coronaviruses downregulate tetherin (16–18).

**Figure 3 –:**
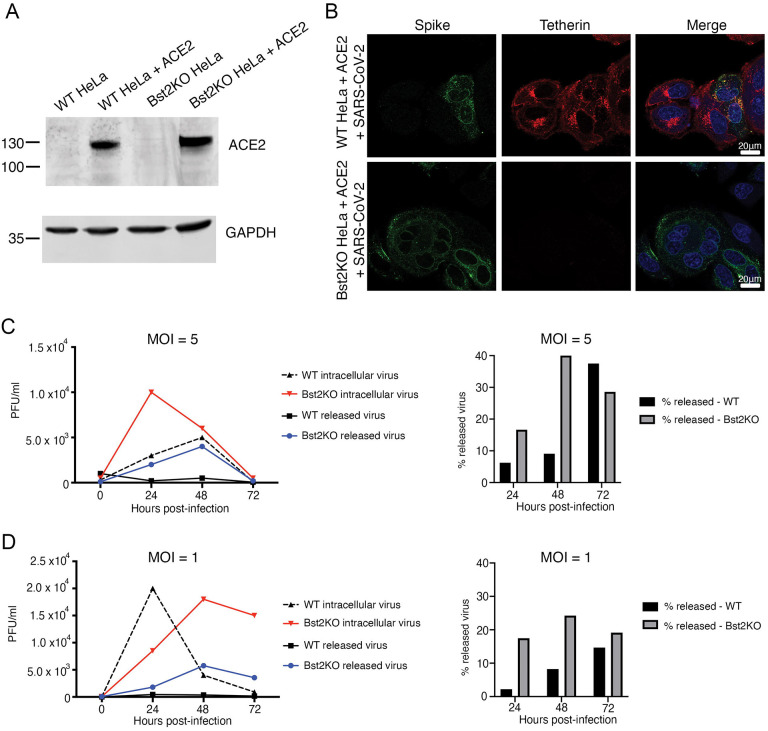
Viral growth curves reveal tetherin loss enhances viral spread. (A) Lentiviral ACE2 was used to generate stable HeLa^Bst2KO^ +ACE2 cells, and ACE2 expression was verified by Western blotting. GAPDH served as a loading control. (B) HeLa^WT^ +ACE2 and HeLa^Bst2KO^ +ACE2 cells were infected with SARS-CoV-2 (MOI 0.5) and fixed at 24 hpi. Cells were stained for spike (green) to demonstrate infection with SARS-CoV-2, and tetherin (red). (C) High MOI viral growth curves were performed by infecting HeLa^WT^ +ACE2 and HeLa^Bst2KO^ +ACE2 cells with SARS-CoV-2 at MOI (5). Titres were measured by plaque assays. (D) Low MOI viral growth curves were performed by infecting HeLa^WT^ +ACE2 and HeLa^Bst2KO^ +ACE2 cells with SARS-CoV-2 at MOI (1). Titres were measured by plaque assays.

To determine whether tetherin plays a functional role in viral tethering, we performed both one-step and multi-step growth curves, to measure both released and intracellular virus production from infected HeLa^WT^+ACE2 and HeLa^Bst2KO^ +ACE2 (Figure 3A). Both HeLa^WT^+ACE2 and HeLa^Bst2KO^ +ACE2 were able to be infected with SARS-CoV-2 (Figure 3B). HeLa^WT^+ACE2 and HeLa^Bst2KO^ +ACE2 cells were infected at the respective MOI and released and intracellular virus was harvested at the indicated time points (see Methods) (Figure 3C, 3D).

The higher MOI of 5, used in the one-step growth curve (Figure 3C), ensures synchronous infection event of 99.3 % of the cells (according to the Poisson distribution). This is predicted to result in synchronised RNA replication, virion assembly and egress, allowing a clear distinction between the various virus life cycle steps, as minimal reinfection should occur. However, this approach results in the vast majority of cells being infected with >1 virus particle. In addition to being less physiologically relevant, this higher viral load may be able to overcome host restriction factors and mask a phenotype. In this case, the released virus titre was clearly higher in the HeLa^Bst2KO^ +ACE2 cells (24 and 48 hpi), evidencing the tetherin-mediated restriction in the HeLa^WT^ +ACE2 cells. However, intracellular virions appeared to accumulate quickly in the HeLa^Bst2KO^ +ACE2 cells at the early time point of 24 hours; whether this is due to a lack of tetherin indirectly allowing enhanced RNA replication or is a by-product of this model system remains to be seen. It is obvious, however, that when the released virions are viewed as a proportion of total infectious particles, the HeLa^Bst2KO^ +ACE2 cells release significantly more than HeLa^WT^ +ACE2 cells at 24 and 48 hpi, due to their inability to tether nascent virions.

By contrast, the multi-step growth curve utilising an MOI of 1 (Figure 3D) has the advantage of being more physiologically relevant and providing a more realistic stoichiometric ratio of viral to host proteins, making it less likely to mask naturally occurring interactions and their resulting phenotypes. However as approximately 37% of the cells will not be infected by the initial inoculum, infection of naïve cells will continue to occur throughout the time course and the viral replication events cannot be presumed to be aligned. In this case, the disparity between HeLa^Bst2KO^ +ACE2 and HeLa^WT^ +ACE2 cells is clear until the final time point of 72 hours, with the HeLa^Bst2KO^ +ACE2 cells continually releasing a higher proportion of virions. Together, these data demonstrate that tetherin acts to limit SARS-CoV-2 infection and that SARS-CoV-2 acts to downregulate tetherin. These data support the notion that tetherin exerts a broad restriction against numerous enveloped viruses, regardless of whether budding occurs at the plasma membrane or within intracellular compartments.

Previously studied coronaviruses, including HCoV-229E and SARS-CoV-1 have been demonstrated to downregulate tetherin (16–18). We next aimed to determine which SARS-CoV-2 protein is responsible for tetherin downregulation.

**Figure 4 –:**
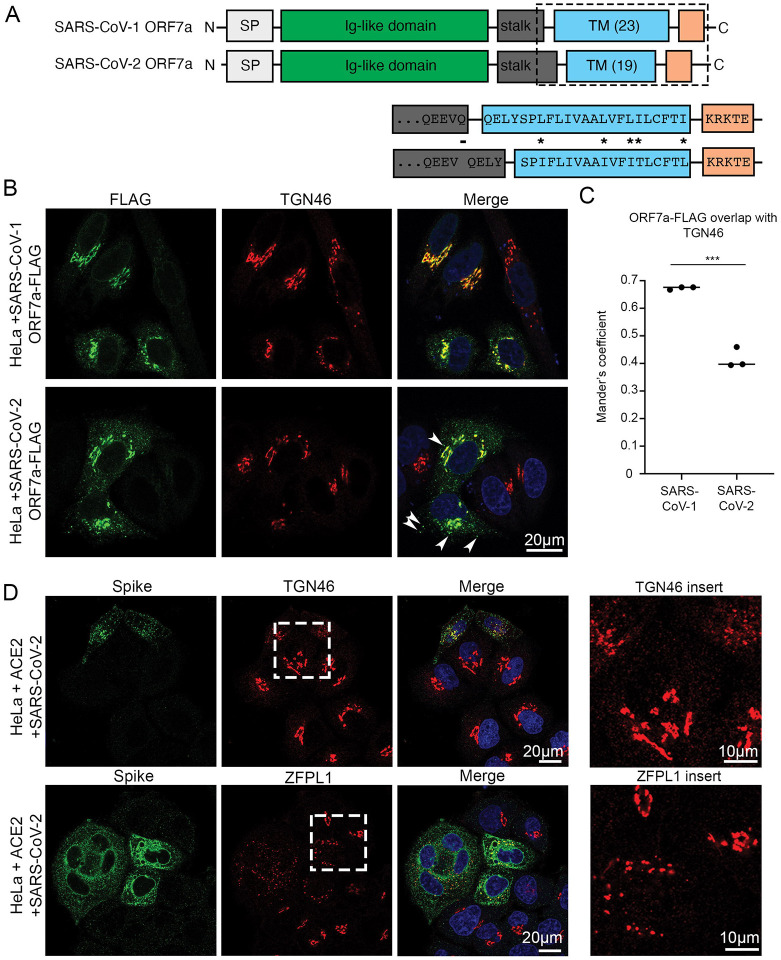
SARS-CoV-2 ORF7a is localized to the TGN, but to a lesser extent than SARS-CoV-1 ORF7a. (A) Schematic diagram to illustrate the domain organization of SARS-CoV-1 ORF7a and SARS-CoV-2 ORF7a. SP = signal peptide. TM = transmembrane domain. Expanded region below shows regions flanking the transmembrane domain with amino acid differences (*) and deletions (–) between SARS-CoV-1 ORF7a and SARS-CoV-2 ORF7a. (B) Representative confocal immunofluorescence images of HeLa cells transiently transfected with SARS-CoV-1 ORF7a-FLAG or SARS-CoV-2 ORF7a-FLAG. SARS-CoV-1 ORF7a-FLAG predominately colocalizes with TGN46 (red), while SARS-CoV-2 ORF7a-FLAG shows additional staining outside that colocalizing with TGN46 (arrowheads). (C) Manders’ coefficients were calculated to measure the ORF7a-FLAG overlap with TGN46. n = 3 independent experiments. Means of each independent experiment are plotted. Two-tailed, unpaired t-tests were performed. *** p < 0.001. (D) SARS-CoV-2 infected HeLa^WT^ +ACE2 cells display fragmentation of Golgi markers. HeLa^WT^ +ACE2 cells were infected with SARS-CoV-2 (MOI 0.5) and fixed at 24 hpi. Infected cells were identified by spike staining (green) and cells were costained with Golgi markers TGN46 (top) and ZFPL1 (below). Areas of TGN46 and ZFPL1 are enlarged (right) to highlight Golgi fragmentation in SARS-CoV-2 infected cells.

The ORF7a protein encodes a single pass type I transmembrane domain containing protein that is localised predominately to the Golgi. SARS-CoV-2 ORF7a protein is one amino acid shorter than its SARS-CoV-1 homologue (SARS-CoV-1 – 122; SARS-CoV-2 – 121 amino acids) (Figure 4A). SARS-CoV-2 ORF7a protein is predicted to contain a 15 residue N terminal signal peptide, a 79 residue Ig-like beta sandwich fold luminal domain, a transmembrane domain and a 5 residue cytosolic tail. The cytosolic domains between the SARS-CoV-1 and SARS-CoV-2 ORF7a protein are identical (KTKRE), and the KxK sequence is found in numerous Golgi resident proteins where it appears to be required for COPII recognition and ER to Golgi trafficking (25). This extremely short cytosolic tail is not sufficient to retain the SARS-CoV-1 ORF7a protein to the Golgi, and it relies on its transmembrane domain for Golgi retention (26). From SARS-CoV-1 to SARS-CoV-2, the transmembrane domain of the ORF7a protein has acquired 5 mutations, although 4 of these appear to be conservative (isoleucine/leucine swaps). Perhaps more pertinent, the one amino acid deletion found in SARS-CoV-2 versus SARS-CoV-1 ORF7a protein is the deletion of a glycine residue immediately at the start of the transmembrane domain which is predicted to reduce the size of the transmembrane domain (SARS-CoV-1 – 23; SARS-CoV-2 – 19).

To compare the cellular localisation of the SARS-CoV-1 and SARS-CoV-2 ORF7a protein we generated C terminally FLAG-tagged ORF7a constructs previously demonstrated to not alter SARS-CoV-1 ORF7a protein localisation (26). Transient transfection of both SARS-CoV-1 ORF7a-FLAG and SARS-CoV-2 ORF7a-FLAG demonstrated both proteins localise to Golgi compartments, although more-so for SARS-CoV-1 ORF7a than SARS-CoV-2 ORF7a (Figures 4B, 4C). Whilst an increase in cytosolic puncta were observed in SARS-CoV-2 ORF7a transfected cells (arrowheads), Golgi markers were found to be less reticular and fragmentation of Golgi markers was observed (Figure 4B). Stable cell lines were generated, and these cells similarly displayed a loss of normal Golgi morphology (Supplemental figures 3A, 3B). We had previously noted a loss of Golgi cisterna in SARS-CoV-2 infected cells by electron microscopy with the concomitant appearance of DMVs in infected cells (Figures 1D, 2E, 2F). We analysed the morphology of Golgi markers in SARS-CoV-2 infected cells by immunofluorescence, and infected cells displayed fragmented TGN and cis-Golgi markers, TGN46 and ZFPL1 respectively, that phenocopied those observed in ORF7a expressing cells (Figure 4D).

**Figure 5 –:**
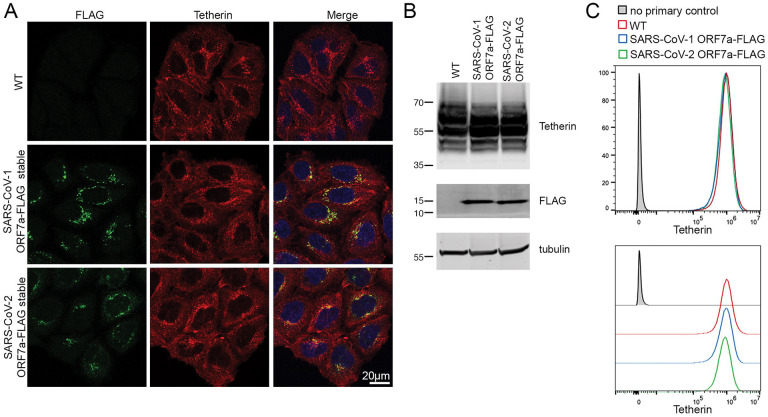
Neither SARS-CoV-1 ORF7a protein, nor SARS-CoV-2 ORF7a protein downregulate tetherin (A) Stable SARS-CoV-1 ORF7a-FLAG and SARS-CoV-2 ORF7a-FLAG HeLa cell lines were generated and tetherin localization was analysed by immunofluorescence. Representative confocal immunofluorescence microscopy images of fixed wild-type, SARS-CoV-1 ORF7a-FLAG or SARS-CoV-2 ORF7a-FLAG HeLa cells were stained using anti-FLAG (green) and anti-tetherin (red) antibodies. (B) Western blotting was performed to confirm the expression of FLAG, and analyse the level of tetherin expression. (C) Flow cytometry was performed on wild-type (red), stable SARS-CoV-1 ORF7a-FLAG (blue) and stable SARS-CoV-2 ORF7a-FLAG (green) HeLa cells to analyse surface tetherin levels.

To determine the levels of tetherin downregulation by ORF7a, HeLa cells stably expressing SARS-CoV-1 ORF7a-FLAG or SARS-CoV2-ORF7a-FLAG were analysed by immunofluorescence, Western blotting and flow cytometry. Neither stable expression of SARS-CoV-1 or SARS-CoV-2 ORF7a caused tetherin to be downregulated (Figure 5A, 5B, 5C). Together, we find that ORF7a does not cause tetherin downregulation, but rather participates in disruption to intracellular protein biosynthesis machineries.

**Figure 6 –:**
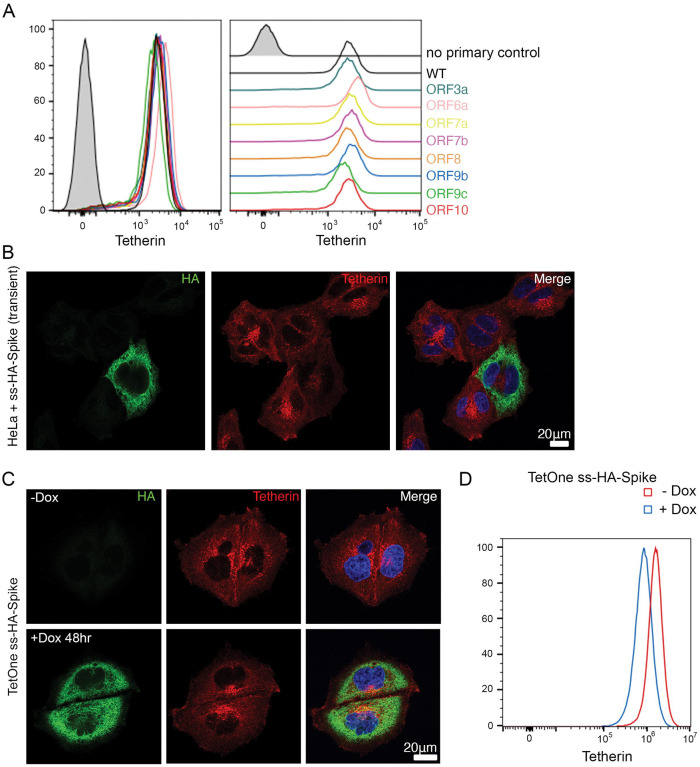
SARS-CoV-2 spike downregulates tetherin (A) A miniscreen was performed to analyse the ability of other SARS-CoV-2 ORFs to downregulate surface tetherin. HeLa cells were transiently transfected with Strep-tagged plasmids encoding: ORF3a-Strep, ORF6-Strep, ORF7a-Strep, Strep-ORF7b, ORF8-Strep, ORF9b-Strep, Strep-ORF9c or ORF10-Strep. 48 hours post transfection, cells were stained for surface tetherin. (B) HeLa cells were transiently transfected with ss-HA-Spike. Transfected cells were identified by anti-HA (green) labelling, cells were costained with an anti-tetherin antibody (red). (C) Stable tetracycline-inducible (TetOne) ss-HA-Spike HeLa cell lines were generated. ss-HA-Spike expression occurs upon induction with Doxycycline. Representative confocal immunofluorescence image showing tetherin loss correlates with HA expression. Anti-HA (green) and anti-tetherin (red). (D) Flow cytometry was performed on tetracycline-inducible ss-HA-Spike cells in resting conditions (no Doxcycline – red) or following induction (plus Doxycycline – blue). Surface tetherin levels were analysed.

We performed a miniscreen to identify SARS-CoV-2 proteins involved in tetherin downregulation by transiently transfecting cells with a number of SARS-CoV-2 2xStrep-tagged ORFs (27). We transiently transfected HeLa cells with: ORF3a-Strep, ORF6-Strep, ORF7a-Strep, Strep-ORF7b, ORF8-Strep, ORF9b-Strep, Strep-ORF9c, ORF10-Strep and confirmed their expression by intracellular flow cytometry using an anti-Strep antibody (Supplemental Figure 4A). Surface tetherin levels were analysed and no significant tetherin downregulation was observed (Figure 6A). Intracellular tetherin localisation was analysed by confocal microscopy and only expression of ORF3a dramatically altered the localisation of intracellular tetherin. ORF3a-Strep transfected cells appeared to accumulate tetherin in intracellular punctate organelles which often colocalised with ORF3a-Strep (Supplemental Figure 4B, untransfected cells shown with asterisk). The SARS-CoV-2 ORF3a gene contains a putative gene in an alternative reading frame, called ORF3c (28). However, the ORF3a-Strep construct used in these experiments has been codon optimised, removing ORF3c. As such, these phenotypes cannot be attributed to ORF3c.

We generated a codon optimised SARS-CoV-2 spike construct containing a HA epitope immediately following the native signal peptide sequence (ss-HA-Spike), rendering the HA tag at the N terminus of the mature protein. Transient transfection of cells with ss-HA-Spike caused a decrease in tetherin as observed by immunofluorescence (Figure 6B), with tetherin being primarily lost from the plasma membrane.

Spike stable cell lines were found to be multinucleated and non-viable, so we generated an inducible ss-HA-Spike cell line using the lentiviral TetOne system to enable transient expression of spike. Tetracycline-inducible ss-HA-Spike stable HeLa cells were generated, and expression of spike was analysed by immunofluorescence following induction through Doxycycline treatment. The level of ss-HA-Spike varied between the population of cells and the higher the level of ss-HA-Spike expression, the larger the tetherin loss (Figure 6C). Even after 48 hours of spike expression, cells formed numerous multi-nucleated syncytia, as reported by others (3)(4, 6). Flow cytometry similarly confirmed that Doxycycline-induced ss-HA-Spike expression led to tetherin downregulation from the surface of cells (Figure 6D).

## Discussion

Tetherin displays a broad ability to restrict numerous families of virus that bud from both the plasma membrane and into intracellular organelles. Tetherin forms homodimers that sit on opposing membranes (e.g., plasma membrane and virus envelope) and these molecules form disulphide bonds between three luminal cysteine residues, linking tetherin molecules together and holding virions to the host plasma membrane. For viruses that undergo membrane envelopment at the plasma membrane, such as HIV-1, are thought to be enriched in tetherin due to tetherin’s GPI-modification which may partition it to cholesterol-rich domains on the plasma membrane. In order for tetherin to restrict SARS-CoV-2, tetherin molecules must be incorporated to the virus during the process of envelopment. Coronaviruses undergo a single envelopment step, and this takes place in modified ERGIC organelles. Whilst tetherin does traffic through these organelles during its biosynthesis, the steady state distribution of tetherin is primarily the plasma membrane and the endocytic pathway (29). Our data support that tetherin molecules become incorporated to SARS-CoV-2 virions during their assembly, and act to restrict virus ‘release’ upon fusion of these organelles with the plasma membrane.

Tetherin is an interferon-stimulated gene (ISG) (10), and many cell types express low levels of tetherin at resting states. Weak type I IFN responses appear as a hallmark of coronavirus infections. SARS-CoV-1 is a poor inducer of type I IFN (30), and SARS-CoV-2 appears even weaker still (31). Interferon has been trailed clinically to treat COVID-19 patients, and tetherin upregulation is likely to be among a series of antiviral proteins which will help combat SARS-CoV-2 spread and infection. By using cell lines that both ubiquitously express high levels of tetherin and cell lines that require induction using interferon, we demonstrate that SARS-CoV-2 mediated tetherin downregulation is not solely due to dysregulation of interferon responses.

Rearrangements of host cellular membranes appears to be a feature of most ssRNA virus infections, including coronavirus, and such rearrangements facilitate the formation of – in the case of coronaviruses - DMVs which act as sites for viral RNA replication. Several coronaviruses induce Golgi fragmentation, including the murine coronavirus mouse hepatitis virus (MHV) (32), avian infectious bronchitis virus (IBV) (33) and SARS-CoV-1 (34). Whilst we have identified SARS-CoV-2 ORF7a as causing Golgi fragmentation, the presence of mutated ORF7a (lacking it’s signal sequence) in so-called ‘Arizona patients’ (35) renders it likely that ORF7a is dispensable for human infection. We did not observe any difference in cell surface tetherin levels, or intracellular distribution of tetherin upon expression of ORF7a. However, we cannot exclude that ORF7a could be perturbing tetherin function by alternative mechanisms, such as inhibiting disulphide bond formation.

Although we did not detect any downregulation of tetherin from the surface of cells upon expression of the SARS-CoV-2 ORFs examined, we did observe the intracellular accumulation of tetherin upon expression of ORF3a. SARS-CoV-1 ORF3a acts as a viroporin at lysosomes, causing a loss of acidification (36), and ORF3a similarly localises to lysosomes (37). The enhanced intracellular tetherin in ORF3a expressing cells likely represents tetherin which has been trafficked to lysosomes but has not been degraded at a normal rate.

SARS-CoV-2 spike has well documented roles in facilitating SARS-CoV-2 viral entry, and in driving the formation of syncytia (38). We also find that SARS-CoV-2 spike causes downregulation of tetherin from the surface of cells. The molecular mechanism behind SARS-CoV-2 spike’s downregulation of tetherin remains unclear.

The convergent evolution displayed by different families of enveloped viruses to downregulate tetherin highlights how important overcoming cellular restriction is to the success of enveloped viral pathogens. Enhancing cellular tetherin levels during such pathogenesis remains a viable and attractive strategy for the management of a multitude of diseases, including COVID-19.

## Materials and methods

### Antibodies

#### Primary antibodies used in the study were:

FLAG rat anti-DYKDDDDK (L5) (BioLegend, WB 1:1000, IF 1:200); rabbit anti-HA antibody (Cell Signalling, C29F4); rat anti-HA antibody (Roche, 3F10); rabbit monoclonal anti-tetherin antibody (Abcam, ab243230, WB 1:2000, IF 1:400, surface EM 1:200); Spike mouse anti-SARS-CoV-2 Spike antibody 1A9 (GeneTex, GTX632604, WB 1:1000, IF 1:300); rabbit anti-TGN46 (abcam, ab50595, 1:300); rabbit anti-ZFPL1 (Sigma-Aldrich, HPA014909, 1:500); rabbit anti-Beta2microglobulin (Dako 1:500); ACE2 (proteintech, 66699, WB 1:1000), Phycoerythrin-conjugated anti-human tetherin antibody (BioLegend, RS38E, FC 1:50), StrepMAB-Classic (IBA LifeSciences, IF 1:500), StrepMAB-Classic DY-549 (IBA LifeSciences, FC 1:500).

#### Secondary antibodies used in this study were:

Goat anti-Mouse IgG Alexa488/555 and Goat anti-rabbit IgG Alexa 488/555 (ThermoFisher) secondaries were used for confocal microscopy.

Goat IRDye 680 anti-mouse, anti-rabbit and Goat IRDye 800 anti-mouse, anti-rabbit antibodies (Li-Cor) were used for Western blotting.

### Cloning

pcDNA6B SARS-CoV-1 and SARS-CoV-2 ORF7a-FLAG constructs were a gift from Peihui Wang (Shandong University, China). To generate stable cell lines, ORF7a-FLAG cDNA fragments were subcloned into pQCXIH retroviral vectors. ss-HA-Spike was generated by cloning an HA epitope plus a Serine-Glycine linker between residues S13 and Q14 of SARS-CoV-2 spike. Following translocation to the ER lumen, cleavage of the signal sequence will render the HA tag at the N terminus of the mature protein. Codon optimised SARS-CoV-2 spike was a gift from Jerome Cattin/Sean Munro (LMB, Cambridge, UK). ss-HA-Spike was originally cloned to pcDNA6B for transient transfection, and subsequently subcloned into pLVX-TetOne. All cloning was verified by Sanger sequencing (GeneWiz).

### Cell lines

A549 cells were a gift from Dr Brian Ferguson, University of Cambridge, UK and were cultured in DMEM supplemented with 10% fetal bovine serum, L-glutamine, and Penicillin/Streptomycin, in 5% CO_2_ at 37 °C. T84 cells were purchased from ATCC and were cultured in DMEM: F-12 medium containing 5% fetal bovine serum, L-glutamine, and Penicillin/Streptomycin, in 5% CO_2_ at 37 °C. HeLa cells were a gift from Prof. Scottie Robinson (CIMR, University of Cambridge, UK) and were cultured in DMEM supplemented with 10% fetal bovine serum, L-glutamine, and Penicillin/Streptomycin, in 5% CO_2_ at 37 °C. Bst2KO HeLa cells were previously described (29). All cells were found free of mycoplasma by immunofluorescence (DAPI), electron microscopy and were tested using MycoAlert Mycoplasma Detection Kit (Lonza).

### Transient transfections

HeLa cells were transfected with 2.5 μg of DNA using HeLa Monster (Mirus Bio). Cells were analysed 48 hours after transfection.

### SARS-CoV-2 ORF miniscreen

HeLa cells were transiently transfected with 2xStrep-tagged SARS-CoV-2 ORF DNA (27) using HeLa Monster (Mirus Bio). After 48 hours transfection, cells were detached and the cell population equally split. Half of the cells were permeabilised and intracellular Strep levels were measured by flow cytometry. Surface tetherin staining was performed on the remaining half of the cells.

### Generation of stable cell lines

#### Lentiviral constructs:

ACE2 stable HeLa and A549 cell lines were generated using the lentiviral pLVX-ACE2-Blasticidin construct from Dr Yohei Yamauchi (University of Bristol). Following transduction, cells were selected with 10 μg/ml blasticidin for 18 days.

TetOne stable cell lines were generated using the pLVX-TetOne system. pLVX-TetOne-Puro-ORF7a-2xStrep and pLVX-TetOne-Puro-2xStrep-ORF9c were a gift from David Gordon (UCSF, USA). pLVX-TetOne-Puro-ss-HA-spike was generated as described above. Following transduction, cells were selected with 1 μg/ml puromycin for 5 days.

HEK293 cells were transfected with lentiviral vectors (pLVX and TetOne) plus packaging plasmids pCMVR8.91 and pMD.VSVG using TransIT-293 (Mirus Bio). Viral supernatants were collected 48 hours after transfection, passed through 0.45 μm filters and recipient cells transduced by ‘spinfection’ – viral supernatants were centrifuged at 1800 rpm in a benchtop centrifuge at 37 °C for 3 hours to enhance viral transduction.

#### Retroviral constructs:

pQCXIH-SARS-CoV-1-ORF7a-FLAG and pQCXIH-SARS-CoV-2-ORF7a-FLAG were transfected to HEK293 cells with the packing plasmids pMD.GagPol and pMD.VSVG using Trans-IT293 (Mirus Bio). Viral supernatants were collected 48 hours after transfection, passed through 0.45 μm filters and recipient cells transduced by ‘spinfection’ – viral supernatants were centrifuged at 1800 rpm in a benchtop centrifuge at 37 °C for 3 hours to enhance viral transduction. Stable cells were selected using 400 μg/ml Hygromycin B for 10 days.

### SARS-CoV-2 infections

HeLa^WT^+ACE2, HeLa^Bst2KO^+ACE2, A549+ACE2 or T84 cells were infected with isolate BetaCoV/ Australia/VIC01/2020 (19), which had been passaged once on Vero cells following receipt from Public Health England. All cells were washed with PBS before being infected with a single virus stock, diluted to the desired MOI with sera-free DMEM (supplemented with 25mM HEPES, penicillin (100 U/mL), streptomycin (100 g/mL), 2mM L-glutamine, 1 % non-essential amino acids). After one hour, the inoculum was removed, and cells washed again with PBS. Infected cells were maintained in DMEM, supplemented with the above-described additions plus 2 % FCS (virus growth media).

For immunofluorescence, cells were plated to glass-bottomed 24-well plates (E0030741021, Eppendorf) and infected at an MOI of 0.5 and incubated for 24 hours, after which plates were submerged in 4% PFA/PBS for 20 min.

For conventional electron microscopy, cells were plated to plastic Thermanox (Nunc) coverslips in 24-well plates and infected at an MOI of 0.5 and incubated for 24 hours, after which plates were submerged in 2% PFA / 2.5% glutaraldehyde / 0.1M cacodylate buffer for 20 minutes.

For surface labelling immunoEM, cells were plated to Thermanox coverslips, infected at an MOI of 0.5 and incubated for 24 hours, and fixed with 4% PFA / 0.1M cacodylate buffer for 20 minutes.

### Conventional electron microscopy

Cells were fixed (described above) before being washed with 0.1M cacodylate buffer. Cells were stained using 1% osmium tetroxide + 1.5% potassium ferrocyanide for 1 hour before staining was enhanced with 1% tannic acid / 0.1M cacodylate buffer for 45 minutes. Cells were washed, dehydrated and infiltrated with Epoxy propane (CY212 Epoxy resin:propylene oxide) before being embedded in Epoxy resin. Epoxy was polymerised at 65 °C overnight before Thermanox coverslips were removed using a heat-block. 70nm sections were cut using a Diatome diamond knife mounted to an ultramicrotome. Ultrathin sections were stained with UA Zero (Agar scientific) and lead citrate. An FEI Tecnai transmission electron microscope at an operating voltage of 80kV was used to visualise samples, mounted with a Soft Imaging System Megaview III digital camera.

### Surface immunogold labelling

To enable luminal surface epitopes to be labelled, cells were fixed with 4% PFA / 0.1 M cacodylate. They were washed with 0.1 M cacodylate buffer before being blocked with 1% BSA/PBS. Coverslips were inverted over drops of either rabbit anti-tetherin or rabbit anti-spike antibodies diluted in 1% BSA/PBS. Coverslips were washed before being incubated with protein A gold. Following gold labelling, cells were re-fixed using 2% PFA / 2.5% glutaraldehyde / 0.1 M cacodylate before being processed for conventional electron microscopy as described above.

### Immunofluorescence

Cells were grown on glass coverslips and fixed using 4% PFA/PBS. Cells were quenched with 15 mM glycine/PBS and permeabilised with 0.1% saponin/PBS. Blocking and subsequent steps were performed with 1% BSA, 0.01% saponin in PBS. Cells were mounted on slides with mounting medium containing DAPI (Invitrogen). Cells were imaged using a LSM700 confocal microscope (63×/1.4 NA oil immersion objective; ZEISS).

### Immunofluorescence colocalisation analysis

Appropriate threshold values were manually applied to each channel and the Manders’ overlap coefficient between two channels was quantified using the JACoP plugin of ImageJ Fiji software (39). The same threshold values were applied to all the cells quantified. At least 54 cells per condition and from 3 independent experiment were analysed.

### Western blotting

Tetherin blots were performed using Laemmli sample buffer and run in non-reducing conditions as previously described (23). For all other blots, lysates were mixed with 4x NuPage LDS sample buffer (ThermoFisher). Gels were loaded to NuPage 4–12% Bis-Tris precast gels (ThermoFisher) and transferred to PVDF membranes before being blocked using 5% milk / PBS / 0.1% Tween. Primary antibodies and secondary antibodies were diluted in PBS-tween. Blots were imaged using a Odyseey CLx (Li-Cor).

### Virus Growth Curves

Multiple subconfluent T25 flasks of WT HeLa +ACE2 and KO HeLa +ACE2 cells were each infected with a single stock of SARS-CoV-2, at an MOI of 5 and 1, for the one-step and multi-step growth curves, respectively. After one hour of infection, cells were washed with PBS and maintained in 5mL virus growth media until harvest. One flask was harvested at each time point (0-, 24-, 48- and 72-hours post-infection). At each time point, the supernatant (containing released virions) was collected, clarified and stored at −80°C. The cell monolayer was scraped into 2mL PBS and subjected to three freeze-thaw cycles to release intracellular virions. Following clarification, the cell debris was discarded, and the remaining supernatant was stored at −80 °C. The infectious titre of all virus samples was determined by plaque assay.

### Plaque assays

Plaque assays were performed as previously described for SARS-CoV-1, with minor amendments (40, 41). Briefly, subconfluent Vero cells in 6-well plates were infected with serial dilutions of the virus sample, diluted in sera-free media, for one hour with constant rocking. After removal of the inocula and washing with PBS, 3mL of 0.2% agarose in virus growth media was overlaid and the cells were incubated at 37°C for 72 hours. At this time the overlay media was removed, cells were washed with PBS and fixed with 10% formalin, before being stained with toluidine blue. Plaques were counted manually.

### Flow cytometry

Cells were gently trypsinised, and surface stained for flow cytometry in PBS with 0.5 % BSA + 1 mM EDTA (FACS buffer) for 30 min on ice. Samples were acquired on a 4 laser Cytoflex S (Beckman coulter, 488 nm, 640 nm, 561 nm, 405 nm).

## Supplementary Material

Supplement 1 **Figure S1** (A) SARS-CoV-2 infected HeLa^WT^ +ACE2 cells (MOI 0.5) were fixed at 24 hpi and stained for spike (green) and tetherin (red). Infected cells display reduced tetherin levels, and broad loss of tetherin from the plasma membrane. Where cell surface tetherin remains, it is often clustered with spike staining (enlarged, arrows). Uninfected cells shown with asterisk.
(B) HeLa^WT^ +ACE2 cells were infected with SARS-CoV-2 (MOI 0.5) and fixed at 24 hpi. Cells were stained for spike (green) and beta2microglobulin (red). No differences in beta2microglobulin were observed between infected and uninfected cells.

Supplement 2 **Figure S2** (A) Tetherin expression is induced with IFNα in A549 cells. Mock and IFNα treated (1000 U/ml, 24 hours) A549 cells were fixed and stained for tetherin (red) and analysed by confocal immunofluorescence microscopy.
(B) Further examples of SARS-CoV-2 virions in infected T84 cells. Tethered virions were frequently present at the plasma membrane, and in intracellular compartments.

Supplement 3 **Figure S3** SARS-CoV-1 ORF7a-FLAG and SARS-CoV-2 ORF7a-FLAG stable cell lines were labelled with antibodies against FLAG (green), or Golgi markers (red) TGN46 (A) and ZFPL1 (B).

Supplement 4 **Figure S4** (A) To verify SARS-CoV-2 ORFs were expressed in cells, cells were fixed, permeabilized and stained with anti-Strep antibodies and analysed by flow cytometry to identify cells expressing strep-tagged SARS-CoV-2 ORFs.
(B) ORF3a transfected cells displayed intracellular tetherin accumulation. Representative confocal immunofluorescence microscopy image of fixed HeLa cells transiently transfected with SARS-CoV-2 ORF3a-Strep. Anti-Strep (green), anti-tetherin (red), DAPI (blue).

## Highlights

- SARS-CoV-2 downregulates the host restriction factor, tetherin.
- Tetherin loss enhances viral titre and spread.
- SARS-CoV-2 ORF7a protein does not downregulate tetherin, but instead induces Golgi fragmentation.
- Tetherin downregulation is mediated by SARS-CoV-2 spike.

## Abbreviations

ACE2: angiotensin converting enzyme 2
Bst2: bone marrow stromal antigen 2
DMV: double membrane vesicle
ERGIC: endoplasmic reticulum-Golgi intermediate compartments
GPI: glycosylphosphatidylinositol
IFN: interferon
LAMP1: Lysosomal-associated membrane protein 1
SARS: severe acute respiratory syndrome
