# Supplementary figures and images for: Tetherin antagonism by SARS-CoV-2 enhances virus release: multiple mechanisms including ORF3a-mediated defective retrograde traffic
Source: bioRxiv. 2022 Dec 22:2021.01.06.425396. Originally published 2021 Jan 6. Preprint. [Version 2] doi: 10.1101/2021.01.06.425396 (PMC7805449; doi:10.1101/2021.01.06.425396)

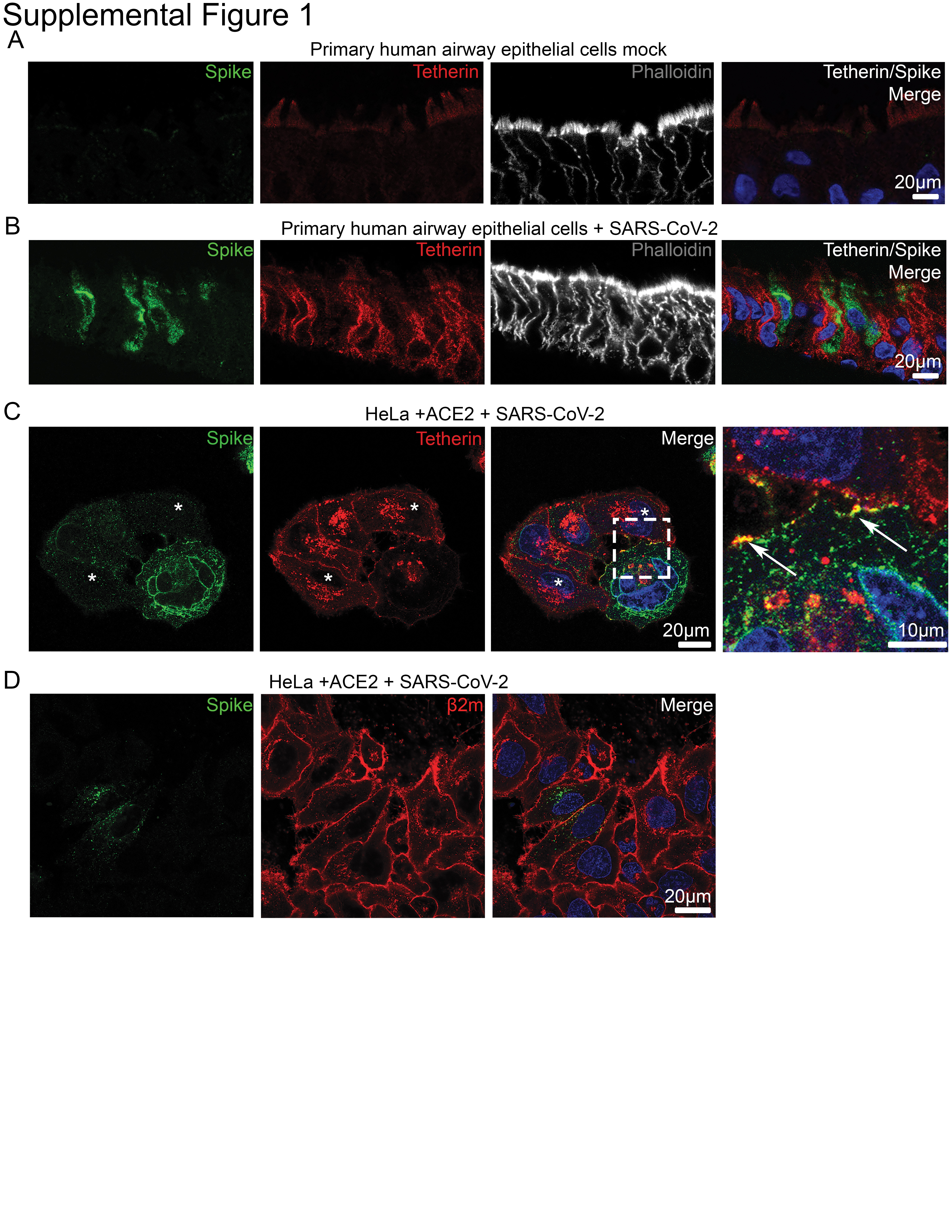

Supplement: Supplement 1 — Figure S1 (A)Mock infected differentiated nasal primary human airway epithelial (HAE) cells were infected and embedded to OCT. Cryostat sections were stained for spike (green), tetherin (red), phalloidin (grey) and DAPI (blue). (B)Differentiated nasal primary human airway epithelial (HAE) cells were infected with SARS-CoV-2 (MOI 0.01). Cells were fixed at 48 hpi and embedded to OCT. Cryostat sections were collected and prepared for confocal microscopy. Sections were immunolabelled with antibodies against SARS-CoV-2 spike (green) - to reveal SARS-CoV-2 infected cells, tetherin (red), and with phalloidin-647 (grey) and DAPI (blue). (C)SARS-CoV-2 infected HeLa+ACE2 cells (MOI 0.5) were fixed at 24 hpi and stained for spike (green) and tetherin (red). Infected cells display reduced tetherin levels, and broad loss of tetherin from the plasma membrane. Where cell surface tetherin remains, it is often clustered with spike staining (enlarged, arrows). Uninfected cells shown with asterisk. (D)HeLa+ACE2 cells were infected with SARS-CoV-2 (MOI 0.5) and fixed at 24 hpi. Cells were stained for spike (green) and beta2microglobulin (red). No differences in beta2microglobulin were observed between infected and uninfected cells. [file media-1.jpg]

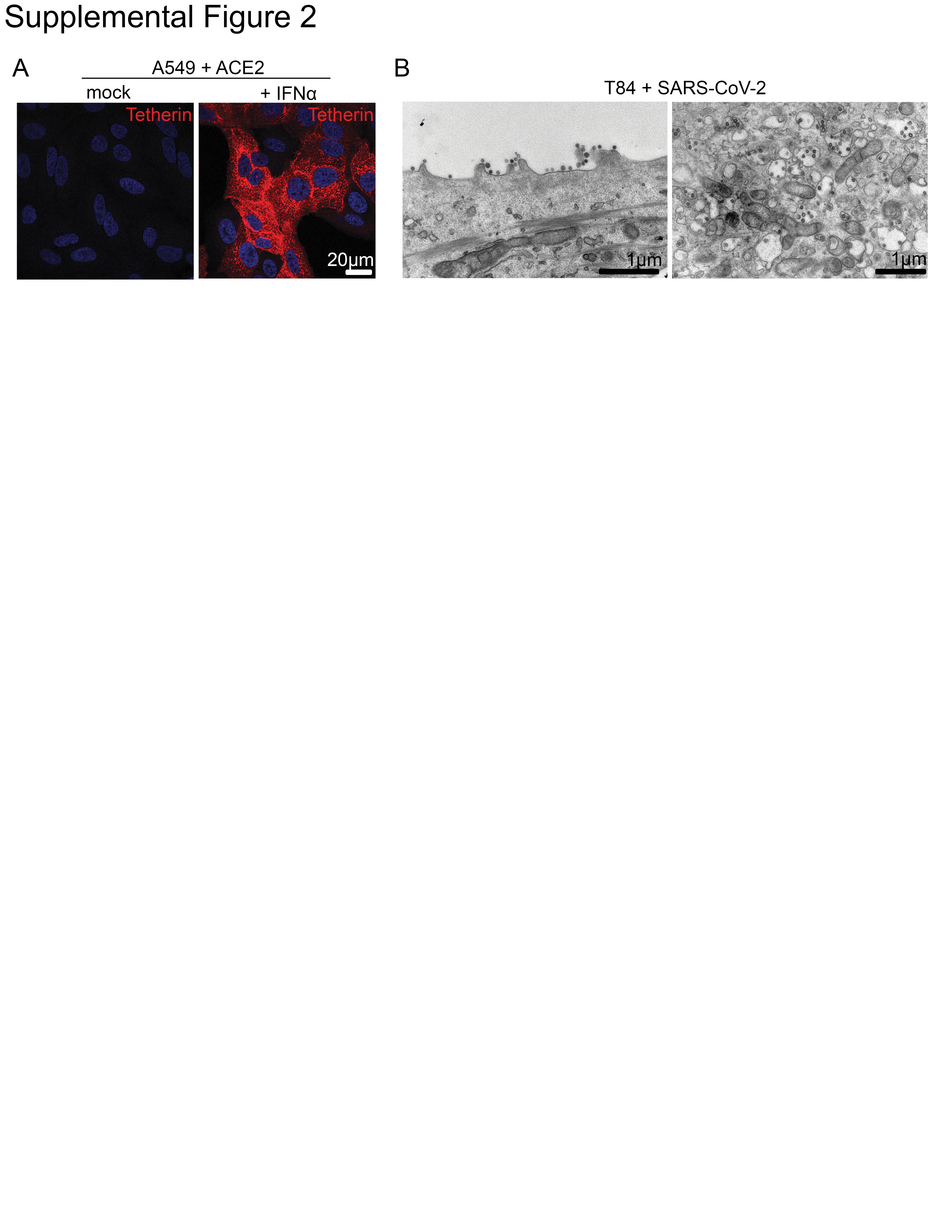

Supplement: Supplement 2 — Figure S2 (A) Tetherin expression is induced with IFNα in A549 cells. Mock and IFNα treated (1000 U/mL, 24 hours) A549 cells were fixed and stained for tetherin (red) and analysed by confocal immunofluorescence microscopy. (B)Additional electron micrographs of SARS-CoV-2 virions in infected T84 cells. T84 cells were infected with SARS-CoV-2 (MOI 0.5) and fixed at 24 hpi. Tethered virions were frequently present at the plasma membrane, and in intracellular compartments. [file media-2.jpg]

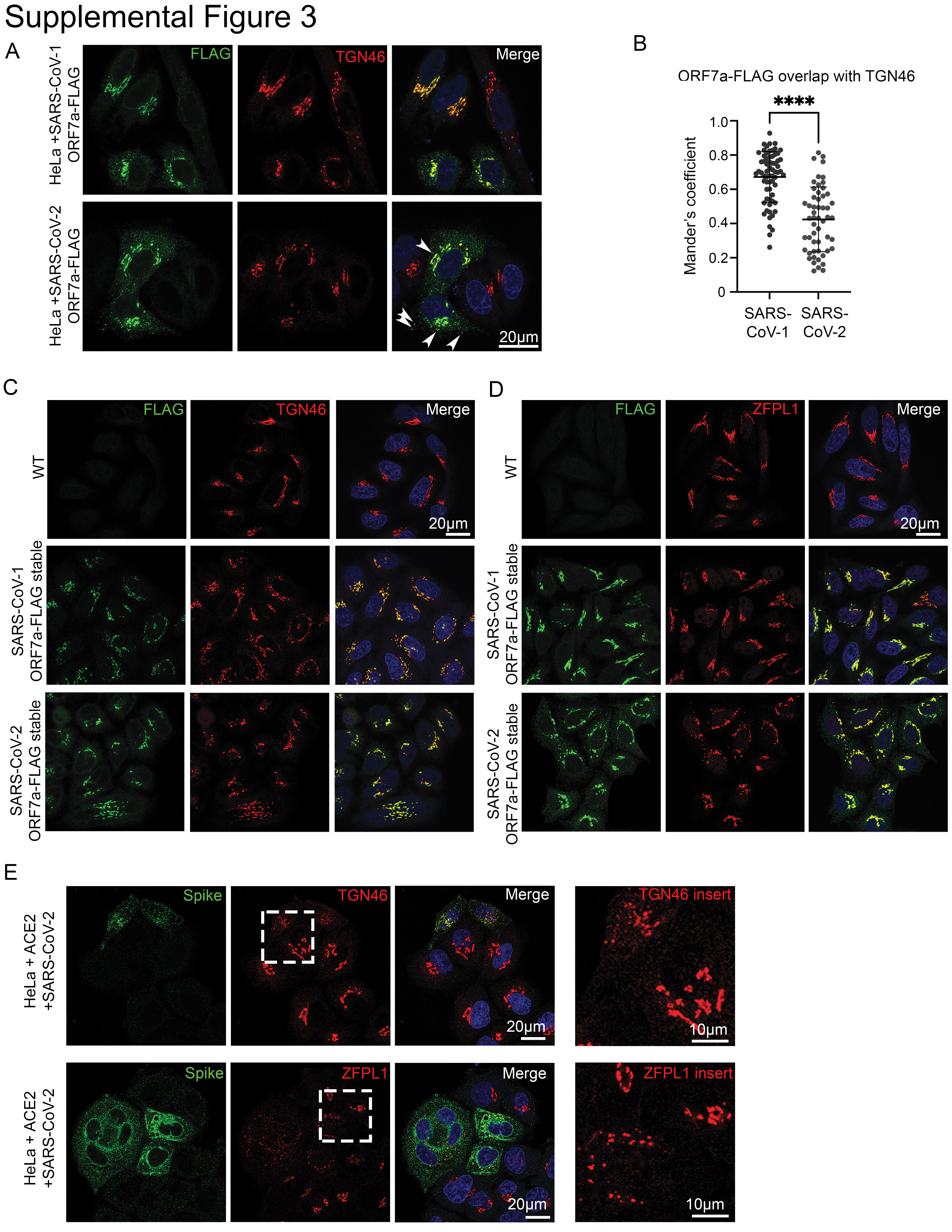

Supplement: Supplement 3 — Figure S3 (A) Representative confocal immunofluorescence images of HeLa cells transiently transfected with SARS-CoV-1 ORF7a-FLAG or SARS-CoV-2 ORF7a-FLAG. SARS-CoV-1 ORF7a-FLAG predominately colocalizes with TGN46 (red), while SARS-CoV-2 ORF7a-FLAG shows additional staining outside that colocalizing with TGN46 (arrowheads). (B)Manders’ coefficients were calculated to measure the ORF7a-FLAG overlap with TGN46. At least 54 cells per condition from three independent experiments were analysed. All data points, mean and standard deviation are plotted. Two-tailed, unpaired t-tests were performed. **** p < 0.0001. (C)SARS-CoV-1 ORF7a-FLAG and SARS-CoV-2 ORF7a-FLAG stable cell lines were labelled with antibodies against FLAG (green), or the trans-Golgi marker TGN46 (red) (D)As in (C) but with the cis-Golgi marker ZFPL1 (red). (E)SARS-CoV-2 infected HeLa+ACE2 cells display fragmentation of Golgi markers. HeLa+ACE2 cells were infected with SARS-CoV-2 (MOI 0.5) and fixed at 24 hpi. Infected cells were identified by Spike staining (green) and cells were costained with Golgi markers TGN46 (top) and ZFPL1 (below). Areas of TGN46 and ZFPL1 are enlarged (right) to highlight Golgi fragmentation in SARS-CoV-2 infected cells. [file media-3.jpg]

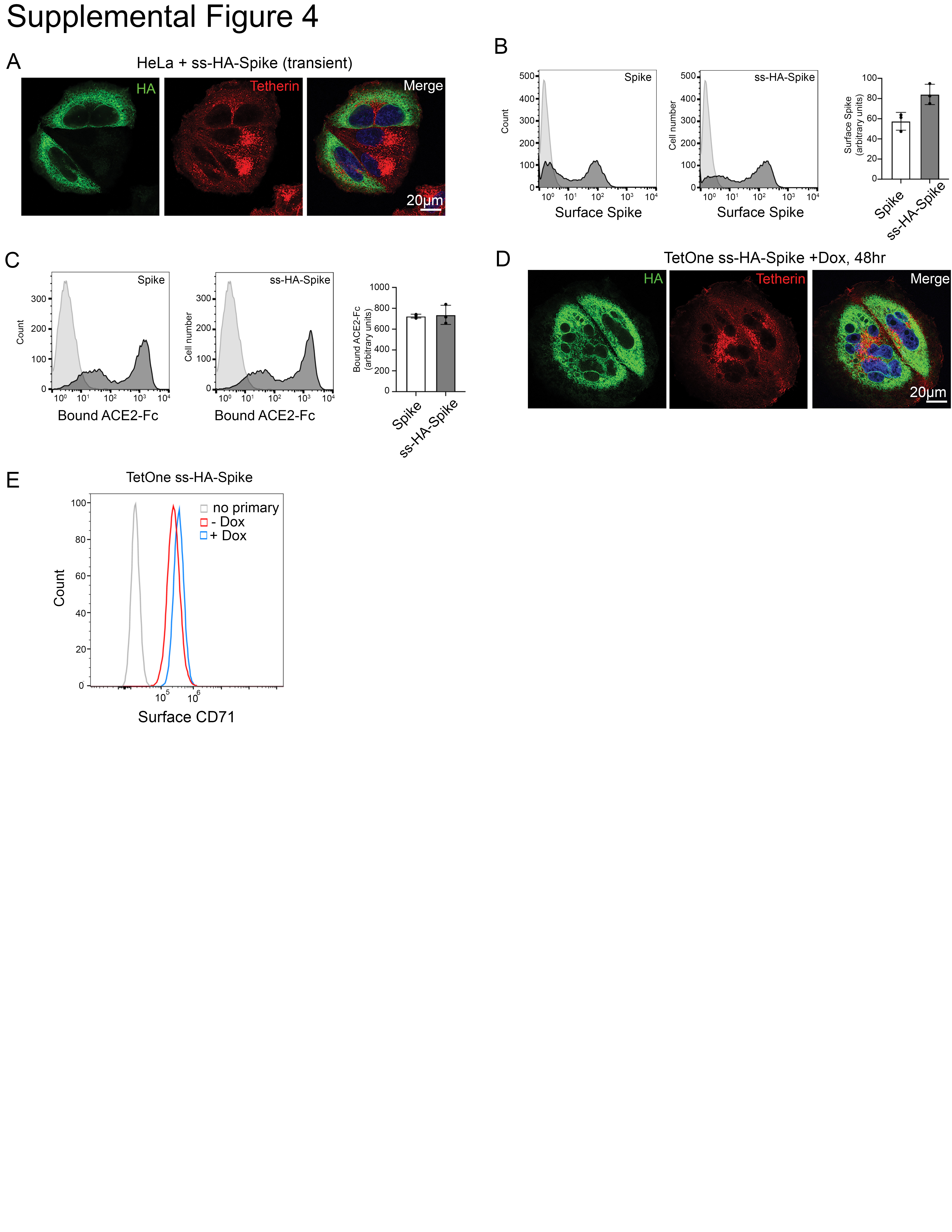

Supplement: Supplement 4 — Figure S4 (A)HeLa cells were transiently transfected with plasmids encoding ss-HA-Spike. 48 hours post transfection, cells were fixed and stained with antibodies against anti-HA (green) and anti-tetherin (red), and DAPI (blue). (B)HEK-293T cells were transfected with the indicated constructs and their surface levels determined by flow cytometry using an antibody directed against the S2 subunit of the Spike protein. The light grey trace shows the non-transfected, unstained control and the dark grey trace the sample expressing the Spike construct. The mean fluorescent intensity was calculated for each sample and plotted. Error bars show the standard deviation for three biological repeats. (C)HEK-293T cells were transfected with the indicated constructs and incubated with tissue culture supernatant containing soluble ACE2 (ACE2-Fc) and the amount of ACE2 binding determined by flow cytometry. The light grey trace shows the non-transfected control sample and the dark grey trace the sample expressing the Spike construct. The mean fluorescent intensity was calculated for each sample and plotted. Error bars show the standard deviation for three biological repeats. (D)TetOne ss-HA-Spike cells were incubated with Doxycycline for 48 hours. Cells were fixed and stained for HA (green), tetherin (red) and with DAPI (blue) to show the presence of multinucleated cells. (E)Flow cytometry was performed on mock or Doxycycline treated (48 hour) TetOne ss-HA-Spike cells to analyse surface CD71 levels. [file media-4.jpg]

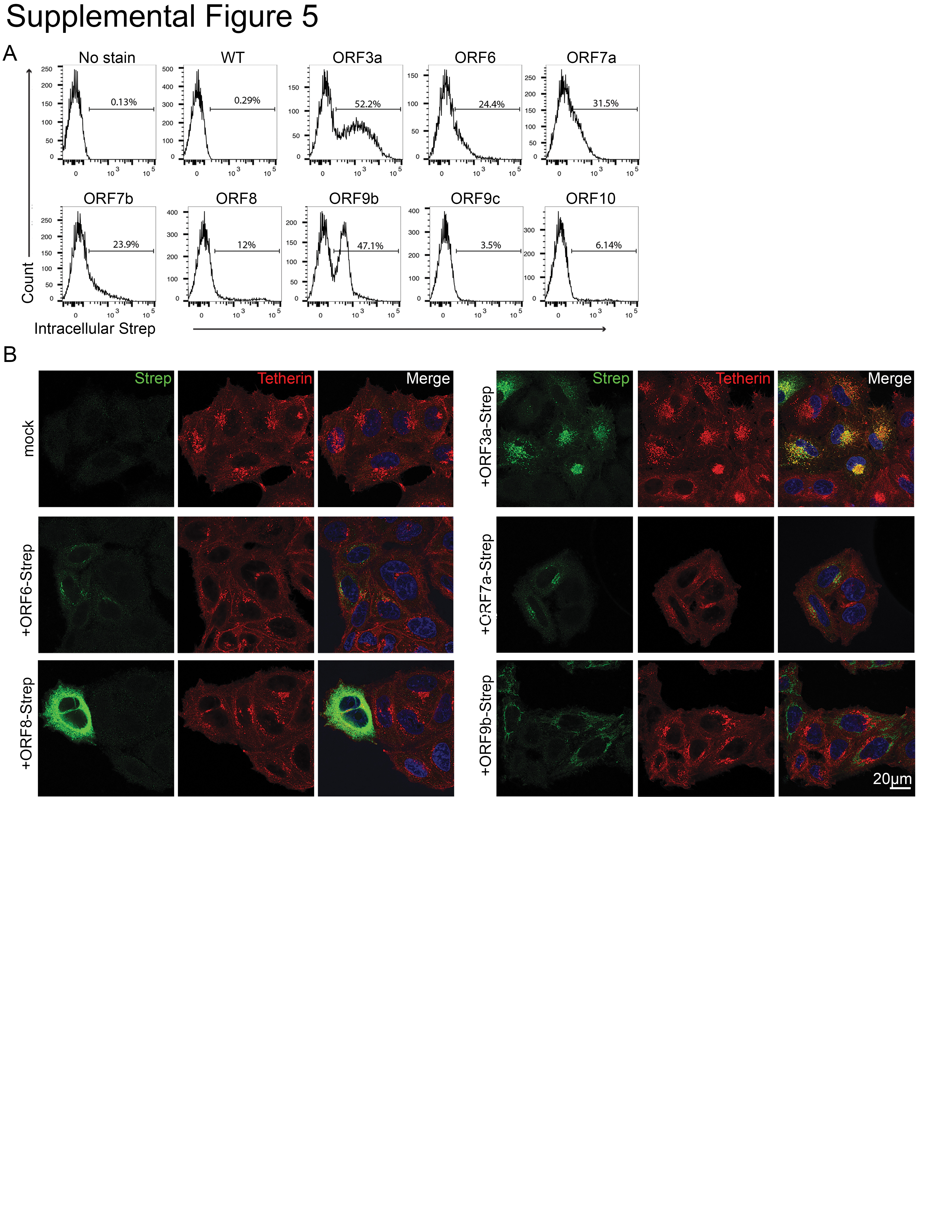

Supplement: Supplement 5 — Figure S5 (A)SARS-CoV-2 ORFs were transiently expressed in HeLa cells. Cells were fixed, permeabilized and stained with anti-Strep antibodies and analysed by flow cytometry to confirm cells expressing strep-tagged SARS-CoV-2 ORFs. (B)Representative confocal immunofluorescence microscopy images of fixed HeLa cells transiently transfected with SARS-CoV-2 ORFs. Anti-Strep (green), anti-tetherin (red), DAPI (blue). [file media-5.jpg]

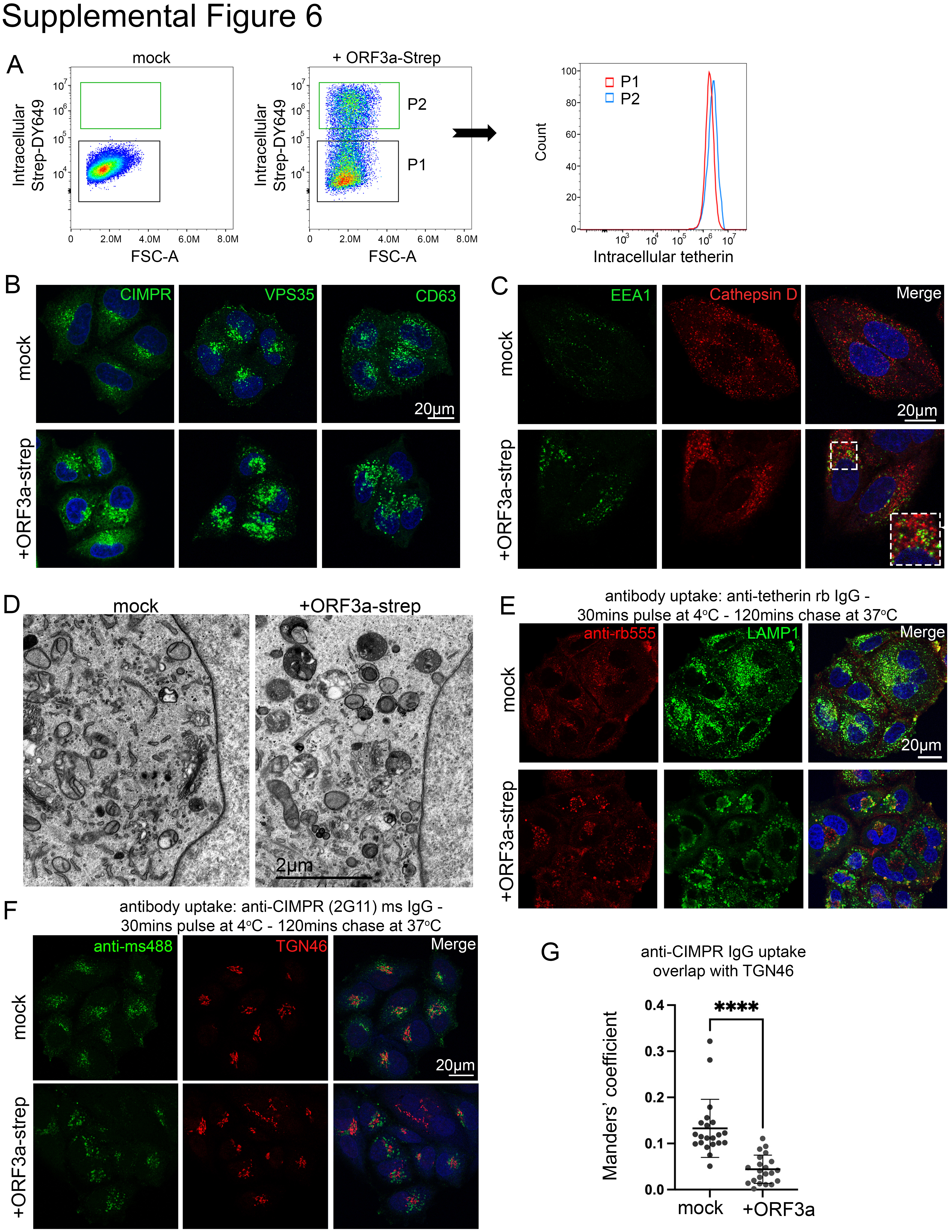

Supplement: Supplement 6 — Figure S6 (A)Intracellular flow cytometry was performed on mock HeLa and transiently transfected ORF3a-Strep HeLa cells to identify Strep positive (transfected) cells. The intracellular levels of tetherin were compared between the Strep negative (untransfected) and Strep positive (transfected) cells. (B)Confocal immunofluorescence microscopy was performed on mock or ORF3a-Strep transfected cells to analyse the distribution of markers CIMPR, VPS35 and CD63. (C)Confocal immunofluorescence microscopy was performed on mock or ORF3a-Strep transiently transfected cells to analyse the distribution of the early and late endolysosomal markers, EEA1 and Cathepsin D. Colocalisation between EEA1 and Cathepsin D could only be observed in ORF3a-Strep transfected cells. Enlarged area shows Cathepsin D within EEA1-positive compartments. (D)Transmission electron microscopy was performed on mock or ORF3a-Strep transiently transfected HeLa cells. Endosomes and lysosomes appeared enlarged and containing non-resolved content in ORF3a-Strep transfected cells. (E)Antibody uptake experiments were performed to follow the fate of endocytosed tetherin. ORF3a-Strep transient transfections were performed 48 hours prior to uptake experiments. Anti-tetherin antibodies were bound to live cells on ice for 30 minutes before a 2 hour chase at 37°C. Cells were fixed and immunolabelled using anti-LAMP1 (green), secondary anti-rabbit555 (red) antibodies, and DAPI (blue). Representative images are shown. (F)Antibody uptake experiments were performed using anti-CIMPR antibodies. ORF3a-Strep transient transfections were performed 48 hours prior to uptake experiments. Anti-CIMPR antibodies (2G11 clone) were bound to live cells on ice for 30 minutes before a 2 hour chase at 37°C. Cells were fixed and immunolabelled using anti-TGN46 (red), secondary anti-mouse (green) antibodies, and DAPI (blue). Representative images are shown. (G)Colocalisation analysis was performed to quantify the Mander’s overlap coeffic [file media-6.jpg]
